# Intraoperative serum lactate levels as a prognostic predictor of outcome for emergency abdominal surgery: a retrospective study

**DOI:** 10.1186/s12893-023-02075-7

**Published:** 2023-06-16

**Authors:** Shinji Sugita, Masashi Ishikawa, Takahiro Sakuma, Masumi Iizuka, Sayako Hanai, Atsuhiro Sakamoto

**Affiliations:** 1 Department of Anesthesiology and Pain Medicine, Graduate School of Medicine, Nippon Medical School, 1-1-5 Sendagi, Bunkyo-ku, Tokyo, 113-8602 Japan; 2 Department of Anesthesiology, Nippon Medical School Musashi-Kosugi Hospital, 1-383 Kosugi-cho, Nakahara-ku, Kawasaki-shi, Kanagawa, 211-8533 Japan; 3 Department of Anesthesia, Urasoe General Hospital, 4-16-1 Iso, Urasoe-shi, Okinawa, 901-2132 Japan; 4 Department of Anesthesiology, Keiyu Hospital, 3-7-3 Minatomirai, Nishi-ku, Yokohama-shi, Kanagawa, 220-8521 Japan

**Keywords:** Hyperlactatemia, Retrospective study, Prognosis, Emergency gastrointestinal surgery

## Abstract

**Background:**

The relationship between intraoperative lactate levels and prognosis after emergency gastrointestinal surgery remains unclear. The purpose of this study was to investigate the prognostic value of intraoperative lactate levels for predicting in-hospital mortality, and to examine intraoperative hemodynamic managements.

**Methods:**

We conducted a retrospective observational study of emergency GI surgeries performed at our institution between 2011 and 2020. The study group comprised patients admitted to intensive care units postoperatively, and whose intraoperative and postoperative lactate levels were available. Intraoperative peak lactate levels (intra-LACs) were selected for analysis, and in-hospital mortality was set as the primary outcome. The prognostic value of intra-LAC was assessed using logistic regression and receiver operating characteristic (ROC) curve analysis.

**Results:**

Of the 551 patients included in the study, 120 died postoperatively. Intra-LAC in the group who survived and the group that died was 1.80 [interquartile range [IQR], 1.19–3.01] mmol/L and 4.22 [IQR, 2.15–7.13] mmol/L (P < 0.001), respectively. Patients who died had larger volumes of red blood cell (RBC) transfusions and fluid administration, and were administered higher doses of vasoactive drugs. Logistic regression analysis showed that intra-LAC was an independent predictor of postoperative mortality (odds ratio [OR] 1.210, 95% CI 1.070 –1.360, *P* = 0.002). The volume of RBCs, fluids transfused, and the amount of vasoactive agents administered were not independent predictors. The area under the curve (AUC) of the ROC curve for intra-LAC for in-hospital mortality was 0.762 (95% confidence interval [CI], 0.711–0.812), with a cutoff value of 3.68 mmol/L by Youden index.

**Conclusions:**

Intraoperative lactate levels, but not hemodynamic management, were independently associated with increased in-hospital mortality after emergency GI surgery.

**Supplementary Information:**

The online version contains supplementary material available at 10.1186/s12893-023-02075-7.

## Background

Serum lactate levels can be used as a marker for the imbalance between oxygen supply and demand resulting from circulatory impairment [1. ]. In critically ill patients, hyperlactatemia often results from tissue hypoxia due to anaerobic glycolysis [2. ]. Thus, hyperlactatemia can also be a predictor of mortality in critically ill patients [3. , 4. ] as well as after surgery [5. ]. For example, Jung et al. reported that for patients admitted for emergency abdominal surgery, the serum lactate level at the time of admission was predictive of the risk of intra-abdominal infection after surgery [6. ], whereas Crough-Brown et al. demonstrated that the peak serum lactate level within 24 h after emergency gastrointestinal (GI) surgery was predictive of in-hospital mortality [7. ]. Similarly, postoperative serum lactate levels have been shown to be a useful predictor of early outcomes and mortality after surgical treatment of colorectal perforations [8. ]. However, the relationship between intraoperative lactate levels and prognosis after emergency GI surgery remains unclear. Intraoperative lactate levels are expected to vary depending on several factors such as preoperative patient status, the type of surgical procedure, and the degree of hemodynamic management needed for hemorrhage. This study aimed to investigate the hypothesis that, among the various intraoperative factors, intraoperative lactate levels would be a significant predictor of in-hospital death after emergency GI surgery.

## Methods

This single-center, retrospective, observational study was conducted at the Nippon Medical School Hospital between 2011 and 2020. This study was approved by the ethics committee of the Nippon Medical School (no. 26–02-427). Informed consent was obtained from our institution’s website as an opt-out option. Data were collected from the medical records.

**Fig. 1:**
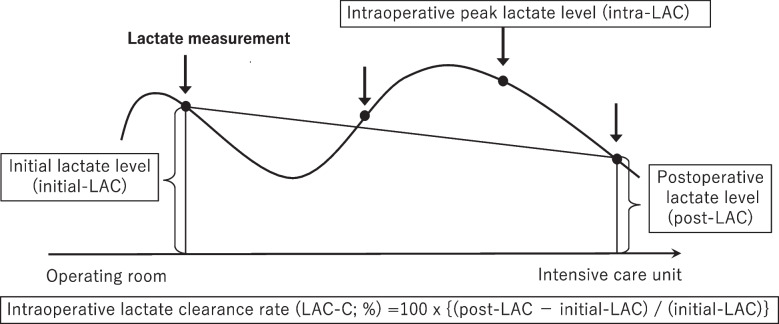
Study design of perioperative lactate measurements. The initial lactate levels (initial LACs) and intraoperative peak lactate levels (intra-LACs) were measured in the operating room. Postoperative lactate levels (post-LACs) were measured upon admission to the intensive care unit. Intraoperative lactate clearance rate (LAC-C) was calculated as follows: LAC-C (%) = 100 × {(post-LA − initial-LAC) / (initial-LAC)}

Patients who had undergone emergency GI surgery except for trauma were enrolled in this study. We only included patients who required admission to intensive care settings and whose intra-and postoperative serum lactate levels were assessed. The criteria for ICU admission were based on the clinician's judgement. First emergency operations were included and cases of second-look procedures, such as an open abdomen strategy after surgery, were excluded to avoid duplication. Cases of laparoscopic surgery were excluded because laparotomy is a well-established approach in our institution, whereas laparoscopic surgery remains a controversial procedure in critically ill patients [9. -11. ]. Blood samples were obtained from arterial catheters at the physician’s discretion. Intraoperative initial and peak lactate levels (initial LACs and intra-LACs, respectively) were selected for analysis (Fig. 1). Postoperative lactate levels (post-LACs) were measured on admission to the intensive care unit after surgery. Intraoperative lactate clearance (LAC-C) was calculated as follows: LAC-C (%) = 100 × {(post-LAC-initial-LAC) / (initial-LAC)}. The primary outcome was overall in-hospital mortality after surgery.

Transfusion was performed according to the Japanese guidelines [12. ]. Briefly, red blood cell (RBC) transfusion was performed with a hemoglobin (Hb) target of 7–8 g/dL for patients with no heart complications, and approximately 10 g/dL for patients with cardiovascular complications, respiratory disease, or cerebrovascular disorders. The intraoperative maximum vasoactive inotrope score (VIS) was calculated as follows: VIS = dopamine (μg/kg/min) + dobutamine (μg/kg/min) + 100 × epinephrine (μg/kg/min) + 100 × norepinephrine (μg/kg/min) + 50 × levosimendan (μg/kg/min) + 10,000 × vasopressin dose (U/kg/min) + 10 × milrinone dose (μg/kg/min) [13. ].

Subsequently, multivariable analyses with logistic regression were performed to investigate whether intra-LAC could be an independent factor for postoperative mortality. The following variables were selected based on previous reports: sex [14. ], age [15. ], surgery for intestinal ischemia/necrosis [16. ], pre-existing ischemic heart disease [17. ], preoperative Hb level [18. ], and sequential organ failure assessment (SOFA) score [6. ]. Intraoperative factors that could influence lactate levels were also selected as follows: maximum intraoperative VIS [13. , 19. ], total amount of intraoperative fluid administration [20. , 21. ], total amount of RBC transfusion, and hemorrhage [22. , 23. ]. SOFA score was calculated as a representative of the preoperative physical status [24. , 25. ].

Finally, the ability of intra-LAC to predict mortality was assessed using the area under the curve (AUC) determined from the receiver operating characteristic (ROC) curve and compared with post-LAC and LAC-C. The cutoff value of intra-LAC for postoperative mortality was calculated using the Youden index.

All statistical analyses were performed using EZR, a graphical user interface for R version 1.54 (R open source). More precisely, this is a modified version of the R commander designed to add statistical functions frequently used in biostatistics [26. ]. Continuous variables were reported as medians with interquartile ranges (IQRs) and compared using the non-parametric Mann–Whitney U test. Categorical variables were presented as frequencies (%) and were evaluated using Fisher’s exact test. The AUC of the ROC curves were compared using EZR statistical guide. Statistical significance was set at *P* < 0.05.

## Results

We identified 551 emergency GI surgery cases that met our inclusion criteria over a 10-year observation period (Table 1). Overall, 120 patients died postoperatively, whereas 431 survived, with overall mortality rate of 21.8%.

**Table 1:** Characteristics of study population (*N* = 551)

*Demographics*	Survival (*N* = 431)	Mortality (*N* = 120)	*P* value
a) Patient characteristics			
- Sex (male, %)	276 (64.0)	58 (48.3)	0.002
- Age (years. old)	71 [61, 79]	79 [72, 84]	< 0.001
Pre-existing disease (%)	325 (75.4)	97 (80.8)	0.268
- CNS (%)	37 (8.6)	12 (10.0)	0.591
- Cerebrovascular Disease (%)	27 (6.3)	8 (6.7)	0.834
- Cardiovascular (%)	199 (46.2)	72 (60.0)	0.01
- Ischemic heart disease (%)	38 (8.8)	23 (19.2)	0.003
- Others (%)	186 (43.2)	61 (50.8)	0.147
- Arrythmia (%)	40 (9.3)	14 (11.7)	0.487
- Respiratory (%)	46 (10.7)	7 (5.8)	0.16
- Acute (%)	9 (2.1)	1 (0.8)	0.698
- Chronic (%)	27 (6.3)	4 (3.3)	0.268
- Liver (%)	3 (0.7)	5 (4.2)	0.014
- Liver cirrhosis (%)	2 (0.5)	3 (2.5)	0.072
- Kidney (%)	27 (6.3)	15 (12.5)	0.031
- Hemodialysis (%)	21 (4.9)	12 (10.0)	0.049
b) Preoperative information			
*Etiology*			
- Upper GI perforation (%)	90 (20.9)	14 (11.7)	0.025
- Lower GI perforation (%)	160 (37.1)	41 (34.2)	0.593
- Obstruction/strangulation (%)	101 (23.4)	19 (15.8)	0.081
- Intestinal ischemia/necrosis (%)	42 (9.7)	45 (37.5)	< 0.001
- Anastomotic leakage (%)	27 (6.3)	3 (2.5)	0.169
- GI hemorrhage (%)	10 (2.3)	2 (1.7)	1
- Other GI disease (%)	19 (4.4)	4 (3.3)	0.798
*Blood*			
- Hemoglobin (g/dL)	12.4 [10.6, 14.4]	10.7 [9.1, 13.1]	< 0.001
- WBC (/dL)	10,100 [5600, 14450]	9450 [4950, 14175]	0.552
- Platelet (10^4/dL)	22.0 [16.7, 29.3]	14.3 [8.3, 22.9]	< 0.001
- Serum creatinine (mg/dL)	0.93 [0.64, 1.8]	1.53 [0.87, 2.38]	< 0.001
- Bilirubin (mg/dL)	0.7 [0.5, 1.1]	1.0 [0.6, 2.1]	< 0.001
- CRP (mg/dL)	8.4 [0.84, 21.7]	10.4 [4.1, 19.3]	0.177
General conditions			
- SOFA score (points)	2 [1, 5]	8 [5,11]	< 0.001
- Glasgow Coma Scale (points)	15 [14,15]	13 [8,15]	< 0.001
- Shock (%)	80 (18.6)	74 (61.7)	< 0.001
- Mechanical ventilation (%)	64 (14.8)	60 (50.0)	< 0.001
- P/F ratio (mmHg)	403 [317, 486]	359 [230, 473]	0.006

Continuous variables are reported as medians with interquartile ranges, and categorical variables are presented as frequencies (%). CNS, central nervous system

Continuous variables were reported as medians with interquartile ranges, and the Mann–Whitney U test was used for analysis. Categorical variables are presented as frequencies (%) and were analyzed using Fisher’s exact test. Shock was defined as systolic blood pressure < 90 mmHg upon arrival in the operating room or as the need for inotropes or vasopressors. *GI* Gastrointestinal, *WBC* White blood cell, *CRP* C-reactive protein, *SOFA* Sequential organ failure assessment, *P/F* PaO_2_/FiO_2_

Among the patients who died in-hospital there was a higher proportion of males compared with the patients who survived (64.0% vs. 48.3%, *P* = 0.002). The patients who died in hospital were older (79 [IQR, 72–84] vs. 71 [IQR, 61–79] years old, *P* < 0.001) than the survival group, and had a higher proportion of pre-existing ischemic heart disease (19.2% vs. 8.8%, *P* = 0.003), liver disease (4.2% vs. 0.7%, *P* = 0.014), and kidney disease (12.5% vs. 6.3%, *P* = 0.031). Patients who died in hospital had a lower proportion of surgery for upper GI perforation (11.7% vs. 20.9%, *P* = 0.025) and a higher proportion of surgery for intestinal ischemia/necrosis (37.5% vs. 9.7%, *P* < 0.001) compared with survivors. The patients who died had lower hemoglobin levels (10.7 [IQR, 9.1–13.1] g/dL vs. 12.4 [IQR, 10.6–14.4] g/dL, *P* < 0.001) and higher SOFA scores (8 [IQR, 5–11] vs. 2 [IQR, 1–5] points, *P* < 0.001). With regard to patient condition, the mortality group had a higher frequency of preoperative shock (61.7% vs. 18.6%, *P* < 0.001) and management with mechanical ventilation (50.0% vs. 14.8%, *P* < 0.001).

Table 2 presents a comparison of intraoperative hemodynamic management between the groups. The operative time did not differ significantly between the groups (patients who died vs. those who survived:137 [IQR 96–188] min vs. 135 [IQR 100–189] min, *P* = 0.887). The patients who died had higher volumes of hemorrhage (95 mL [IQR 2–802 mL] vs. 40 mL [IQR 0–254 mL], *P* = 0.001) and were administered higher volumes of fluid (4185 [IQR 2353 –6679] mL vs. 3200 [IQR 2200–4710], *P* = 0.002), RBC transfusion (4 [IQR, 0–6] units vs. 0 [IQR, 0–2] units, *P* < 0.001), and HES/colloid administration (225 [IQR, 0–500] units vs. 0 [IQR, 0–500] units, *P* = 0.044) than the patients who survived. Lower urine output (113 [IQR, 0–300] vs. 245 [IQR, 100–400] points, *P* < 0.001) was observed in the patients who died. The patients who died had a higher VIS (22 [IQR, 10–43] vs. 0 [IQR, 0–15] points, *P* < 0.001) than the patients who survived. The initial, intra-LAC and post-LAC levels were higher in the patients who died (3.46 [IQR, 1.84–6.26] vs. 1.56 [IQR, 1.03–2.64] mmol/L, *P* < 0.001, 4.22 [IQR, 2.15–7.13] vs. 1.80 [IQR, 1.19–3.01] mmol/L, *P* < 0.001 and 3.72 [IQR, 1.97–7.36] vs. 1.70 [IQR, 1.11–2.77] mmol/L, *P* < 0.001, respectively). The LAC-C rate was not significantly different between the groups (2.88 [IQR, -19.0–28.4] vs. 5.78 [IQR, -20.6–39.5], *P* = 0.796).

**Table 2:** Intraoperative managements (*N* = 551)

*Intraoperative managements*	Survival (*N* = 431)	Mortality (*N* = 120)	*P* value
Operation time (min)	135 [00, 189]	137 [96, 188]	0.887
Hemorrhage (mL)	40 [0, 254]	95 [2, 802]	0.001
Total fluid administration (mL)	3200 [2200, 4710]	4185 [2353, 6679]	0.002
- Transfusion, yes (%)	152 (35.3)	96 (80.0)	< 0.001
- Amount of RBC Transfusion (units)	0 [0, 2]	4 [0, 6]	< 0.001
- Crystalloid administration (mL)	2600 [1750, 3900]	2600 [1375, 4725]	0.729
- HES/colloid administration (mL)	0 [0, 500]	225 [0, 500]	0.044
Urine output (mL)	245 [100, 400]	113 [0, 300]	< 0.001
MAX VIS (points)	0 [0, 15]	22 [10, 42]	< 0.001
*Lactate measurement*			
- Initial-LAC (mmol/L)	1.56 [1.03, 2.64]	3.46 [1.84, 6.26]	< 0.001
- Intra-LAC (mmol/L)	1.80 [1.19, 3.01]	4.22 [2.15, 7.13]	< 0.001
- Post-LAC (mmol/L)	1.70 [1.11, 2.77]	3.72 [1.97, 7.36]	< 0.001
- LAC-C (%)	5.78 [-20.6, 39.5]	2.88 [-19.0, 28.4]	0.796

Continuous variables were reported as medians with interquartile ranges, and the Mann–Whitney U test was used for analysis. Categorical variables are presented as frequencies (%) and were analyzed using Fisher’s exact test. *RBC* Red blood cell, *MAX VIS* Maximum vasoactive inotropic score, *initial-LAC* Initial lactate level, *intra-LAC* Intraoperative peak lactate level, *post-LAC* Postoperative lactate level, *LAC-C* Intraoperative lactate clearance

Multivariate analysis using logistic regression revealed that intra-LAC (odds ratio [OR] 1.21, 95% CI 1.07–1.36, *P *= 0.002) was an independent factor to predict in-hospital mortality after surgery (Table 3). Male sex, (OR 0.546, 95% CI 0.309–0.965, *P* = 0.037), older age (OR 1.050, 95% CI 1.020–1.070, *P* = 0.001), intestinal ischemia/necrosis (OR 2.700, 95% CI 1.370 –5.330, *P* = 0.004), preoperative hemoglobin level (OR 0.881, 95% CI 0.786–0.988, *P* = 0.030) and SOFA score (OR 1.230, 95% CI 1.130–1.340, *P* < 0.001) had statistically significant differences between the two groups of patients.

**Table 3:** Result of logistic regression analysis for in-hospital mortality after surgery

Variables	Odds ratio	95% CI	*P* value
Sex, male	0.546	0.309 to 0.965	0.037
Age, (years.old)	1.050	1.020 to 1.070	0.001
Intestinal ischemia/necrosis, yes	2.700	1.370 to 5.33	0.004
Pre-existing ischemic heart disease, yes	2.000	0.970 to 4.130	0.065
Hemoglobin, g/dL	0.881	0.786 to 0.988	0.030
SOFA score, points	1.230	1.130 to 1.340	< 0.001
MAX VIS, points	1.010	0.996 to 1.030	0.137
Total fluid administration, mL	1.000	1.000 to 1.000	0.328
Amount of RBC transfusion, units	1.050	0.946 to 1.160	0.383
Hemorrhage, mL	1.000	1.000 to 1.000	0.624
Intra-LAC, mmol/L	1.210	1.070 to 1.360	0.002

*SOFA* Sequential organ failure assessment, *MAX VIS* Maximum vasoactive-inotropic score, *intra-LAC* Intraoperative peak lactate level, *CI* Confidence interval

**Fig. 2:**
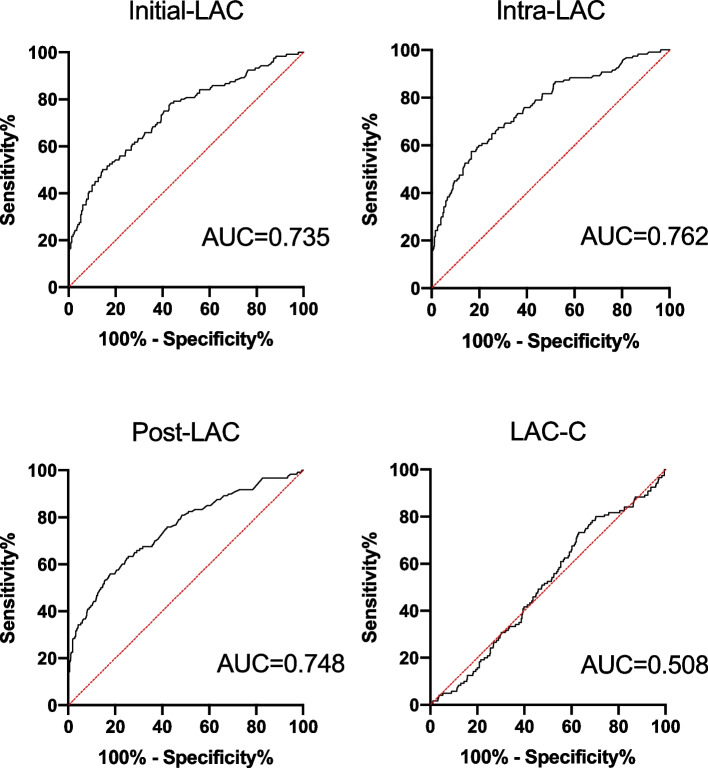
Predictive ability of intraoperative serum lactate levels for hospital mortality (*N* = 551). The AUC of intra-LAC was larger than that of the initial-LAC (*P* = 0.024) and not significantly different from that of post-LAC (*p* = 0.306). The cut-off values of initial LAC, intra-LACand post-LAC for postoperative mortality were 3.58 mmol/L (sensitivity, 0.5; specificity, 0.856), 3.68 mmol/L (sensitivity, 0.575; specificity, 0.833) and 3.33 mmol/L (sensitivity, 0.558; specificity, 0.821), respectively, Youden index. Initial LAC, initial lactate level; intra-LAC, intraoperative peak lactate level; post-LAC, postoperative lactate level; LAC-C, intraoperative lactate clearance; AUC, area under the curve

The AUCs of the initial-LAC, intra-LAC, post-LAC, and LAC-C for in-hospital mortality determined from the ROC curve analysis were as follows: AUC = 0.735, 95% CI, 0.682–0.789; AUC = 0.762, 95% CI, 0.711–0.812; AUC = 0.748, 95% CI, 0.695–0.801; and AUC = 0.508, 95% CI: 0.451–0.564, respectively (Fig. 2). The AUC of intra-LAC was larger than that of initial-LAC (*P* = 0.024) and LAC-C (*P* < 0.001) but did not differ from that of post-LAC (*P* = 0.306). The cutoff value of intra-LAC for postoperative mortality was 3.68 mmol/mL (sensitivity, 0.575; specificity, 0.833), calculated using the Youden index.

## Discussion

In this study, we examined the prognostic value of intraoperative lactate level for outcomes after emergency GI surgery. The main finding of this study was that intraoperative hyperlactatemia was strongly associated with increased mortality after emergency GI surgery.

We performed a logistic regression analysis to investigate whether the intraoperative peak lactate level would be an independent predictor of prognosis. We found that intraoperative peak lactate level could be a prognostic factor for mortality. Several previously reported representative prognostic factors were selected for the multivariate analysis. As critically ill patients sometimes require a large amount of fluid to improve hemodynamic failure, fluid management during surgery can be associated with prognosis. Excessive fluid administration is a risk factor for fluid-related medical interventions, and a high central venous pressure is associated with poor prognosis [20. , 21. ]. Transfusion has also been associated with postoperative complications. Turan et al. reported that massive perioperative transfusion increases the risk of respiratory complications and infectious diseases [22. ]. Nacionales et al. reported that RBC transfusion alters the immune response during sepsis in mice, suggesting that transfusion may lead to poor outcomes in critically ill patients [27. ]. In contrast, the Transfusion Requirements in Septic Shock trial showed that lower and higher Hb thresholds for transfusion in septic shock did not influence mortality or the use of life support [28. ]. American Society of Anesthesiologists Task Force recommend RBC transfusion should be based on cardiopulmonary reserve as well [23. ]. Ischemic heart disease (IHD) is associated with perioperative cardiac events and mortality [17. ]. Intraoperative VIS could reportedly be a predictor of postoperative outcomes in cardiac surgery [13. , 19. ]. We also considered the preoperative condition presented in the severity scoring system, such as the SOFA score, which is an objective score obtained from the calculation of six organ dysfunctions (respiratory, coagulation, liver, cardiovascular, renal, and central nervous systems) [24. ]. Lactate levels can vary intraoperatively depending on various factors, such as the metabolic balance of organs and fluid balance during hemodynamic management. Interestingly, our logistic analysis showed that hemorrhage, amount of RBC transfusion and fluid administration, and VIS were not predictive of outcomes, showing the relevance of lactate measurement during surgery. The measurement of intraoperative lactate levels in patients may be useful as one of the intraoperative strategies.

Several reports have demonstrated the prognostic value of lactate levels in patients with acute gastrointestinal diseases. Kang et al. reported that the postoperative lactate level was a strong predictor of in-hospital mortality in patients who underwent surgery for GI perforation (AUC = 0.771) [29. ]. On the other hand, Jung et al. reported that lactate level (AUC = 0.659) measured in the emergency department in patients with Intra-abdominal infections had a lower predictive value for in-hospital mortality (AUC = 0.795) than SOFA score [6. ]. Our study showed that intraoperative lactate levels were not significantly different from postoperative lactate levels in predicting postoperative in-hospital mortality. Moreover, there was no significant difference between intraoperative lactate levels and SOFA scores (Supplemental Fig. 1). Although our study population was not the same as other studies, our findings suggest that intraoperative peak lactate level may help to predict prognosis.

In critically ill patients, absolute lactate levels and lactate clearance can predict patient outcomes. Haas et al. reported an association between 12-h lactate clearance in patients with severe hyperlactatemia and intensive care unit mortality [30. ]. Lokhandwala et al. reported that a > 20% reduction in lactate levels from baseline at 6 h was associated with in-hospital mortality [31. ]. In addition, the Surviving Sepsis Campaign guidelines of 2016 and 2021 recommend normalizing lactate levels as a therapeutic strategy [32. , 33. ]. However, the intraoperative lactate clearance calculated in our study population was not useful in predicting postoperative mortality. One possible explanation is that our study population included many patients with lactate levels within the normal range (< 2 mmol/L). Another possibility is that the operation time was too short to assess lactate clearance. Since lactate measurement following surgery was not possible in very critical patients because of their early death or other factors, we evaluated intraoperative lactate clearance using postoperative lactate levels in the present study; however, future studies should examine the relationship between perioperative lactate clearance and postoperative management.

### Limitations

Our study has several limitations regarding the interpretation of the results. First, our single-center retrospective study had a small sample size. Second, the effects of confounding factors were not completely minimized in our analysis as we selected patients requiring emergency GI surgery. The complexity of the preoperative health status varied significantly between the cases, which would have influenced preoperative management. Time to surgery also reportedly influences the prognosis of patients with septic shock who require emergency GI surgery [34. ]. As this study included out-of-hospital-onset surgeries as well as in-hospital-onset surgeries (e.g., anastomotic leakage after scheduled GI surgery), the relationship between time-to-surgery and lactate levels could not be evaluated. Third, a lactate measurement protocol was not established in this retrospective study. The peak LACs were not the real peak levels because a continuous lactate monitoring device was not available [35. ]. Finally, the relationship between lactate levels and anesthetic agents was not investigated in this study. Since anesthetic agents can cause dose-related cardiovascular or hemodynamic depression, the dosage of anesthetic agents should be reduced as carefully as possible in patients with hemodynamic instability [36–42]. However, it was difficult to investigate how the anesthetic agents for each surgical stress affected lactate levels. In addition, preoperative sedatives or analgesics might influence intraoperative anesthesia. Patients with or without preoperative mechanical ventilation were included in this study, which might result in differences in the intraoperative dosage of anesthetic agents. Therefore, a well-designed prospective study is required to satisfactorily evaluate the relationship between lactate management and prognosis after emergency surgery.

## Conclusions

In emergency GI surgery, the intraoperative lactate level, but not hemodynamic managements, was independently associated with increased in-hospital mortality. The prognostic value of intraoperative lactate level for in-hospital mortality was comparable to that of postoperative lactate level. Lactate measurement during surgery may be useful; however, the prognostic ability of lactate clearance during surgery was poor. Further studies are needed to investigate intraoperative strategies based on the lactate levels.

## Supplementary Information

**Additional file 1:** **Supplement Figure 1.** Prediction ability of SOFA score for hospital mortality. The AUC of the SOFA score for postoperative mortality was not significantly different from that of intra-LAC but was larger than that of initial LACand post-LAC . The cut-off value of the SOFA score for postoperative mortality was 7 points, calculated using the Youden index. SOFA, sequential organ failure assessment; AUC, area under the curve; intra-LAC, intraoperative peak lactate level; initial-LAC, initial lactate level; post-LAC, postoperative lactate level.

## Data Availability

The datasets used and/or analyzed during the current study are available from the corresponding author upon reasonable request.

## Abbreviations

GI: Gastrointestinal
Intra-LAC: Intraoperative peak lactate level
Initial-LAC: Initial lactate level
Post-LAC: Postoperative lactate level
LAC-C: Intraoperative lactate clearance
RBC: Red blood cell
Hb: Hemoglobin
VIS: Vasoactive inotrope score
OR: Odds ratio
CI: Confidence interval
SOFA: Sequential organ failure assessment
AUC: Area under the curve
ROC: Receiver operating characteristic
IQR: Interquartile range
