# Supplementary material for: Intraoperative serum lactate levels as a prognostic predictor of outcome for emergency abdominal surgery: a retrospective study
Source: BMC Surg. 2023 Jun 16;23:162. doi: 10.1186/s12893-023-02075-7 (PMC10276372; doi:10.1186/s12893-023-02075-7)
Supplement: Supplementary file 1 — Additional file 1: Supplement Figure 1. Prediction ability of SOFA score for hospital mortality. The AUC of the SOFA score for postoperative mortality was not significantly different from that of intra-LAC but was larger than that of initial LACand post-LAC . The cut-off value of the SOFA score for postoperative mortality was 7 points, calculated using the Youden index. SOFA, sequential organ failure assessment; AUC, area under the curve; intra-LAC, intraoperative peak lactate level; initial-LAC, initial lactate level; post-LAC, postoperative lactate level. [file 12893_2023_2075_MOESM1_ESM.docx]

**Supplement Figure 1.**

**Supplement Figure. 1. Prediction ability of SOFA score for hospital mortality (N = 551)**

The AUC of the SOFA score for postoperative mortality was not significantly different from that of intra-LAC (p=0.06) but was larger than that of initial LAC (P=0.010) and post-LAC (P=0.024). The cut-off value of the SOFA score for postoperative mortality was 7 points (sensitivity, 0.65; specificity, 0.861), calculated using the Youden index. SOFA, sequential organ failure assessment; AUC, area under the curve; intra-LAC, intraoperative peak lactate level; initial-LAC, initial lactate level; post-LAC, postoperative lactate level.
